# Sodium Replacement with KCl and MSG: Attitudes, Perception and Acceptance in Reduced Salt Soups

**DOI:** 10.3390/foods12102063

**Published:** 2023-05-20

**Authors:** Jordan C. Walker, Robin Dando

**Affiliations:** 1 College of Human Ecology, Cornell University, Ithaca, NY 14850, USA; 2 Department of Food Science, Cornell University, Ithaca, NY 14853, USA

**Keywords:** sensory, salt, sodium reduction, food

## Abstract

Sodium intake is linked to multiple negative health outcomes, particularly hypertension, the leading cause of premature death globally. Sodium intake levels in human populations are high, due in part to our desire for palatable salty-tasting foods. Two leading salt replacement strategies are the use of potassium chloride (KCl) and monosodium glutamate (MSG), the latter of which still contains some sodium, but both of which can replace some salty taste in foods while reducing net sodium levels. In this report, we employed a trained descriptive sensory panel to optimize saltiness in sodium-reduced aqueous samples using various concentrations of KCl and MSG. Following this, we assessed consumer attitudes to sodium-reduction strategies in a model food, canned soup, known to typically be high in sodium. Finally, in a large consumer test, we verified that these optimized levels of KCl and MSG did not lead to a drop in liking for the reduced-sodium soups with saltiness subsidized in this manner. Our results showed that sodium can be readily reduced in soups by 18% while actually scoring higher in liking, and in some cases being perceived as even more salty tasting, but that consumers are more open to sodium reduction in this manner when sodium replacements are not specifically highlighted, and when percentage sodium reduction is stated over absolute levels.

## 1. Introduction

Although sodium consumption is crucial for human health, overconsumption is linked to multiple poor health outcomes. Consuming excess sodium is correlated with the incidence of hypertension, cardiovascular disease, kidney disease, and gastric cancer [1,2,3,4]. It is accepted that reducing sodium intake lowers blood pressure which can consequently reduce the risk of diseases related to high blood pressure [5].

The effects of sodium intake reduction on blood pressure in normotensive individuals are similar to its effects in hypertensive individuals [5]. There exists some discussion on the efficacy of low-sodium diets on improving health outcomes, with one meta-analysis reporting a U-shaped association between sodium intake and all-cause mortality, with people who have daily intakes of less than 115 mmol and more than 215 mmol having higher mortality than those in the middle of this range [6]. Another systematic review and meta-analysis, however, found no U-shaped association between sodium intake and cardiovascular disease risk, only finding a positive association between the variables [7]. There are also inconsistent findings about the influence of sodium intake on blood lipid profiles. The authors of one review reported that, in both normotensive and hypertensive participants, dietary sodium reduction increased serum cholesterol and triglyceride levels [8], which are associated with increased CVD mortality and all-cause mortality, respectively [9,10]. Yet another study reported that a moderate sodium intake reduction in the range of 50 to 150 mmol per day had no effect on the examined blood lipid profiles [11]. Inconsistencies in findings about the relationship between dietary sodium intake and health risks may stem from varied interpretations of the terms “low sodium” and “high sodium” with respect to diet [12]. Overall, prolonged modest, but not excessive dietary sodium reduction in both hypertensive and normotensive individuals has the potential to benefit public health [5].

The Dietary Guidelines for Americans recommend that adults limit sodium intake to less than 2300 mg per day [13]. Despite this recommendation, the average daily sodium intake of Americans is about 3600 mg [14]. Sodium intake most commonly comes in the form of table salt, specifically sodium chloride (NaCl) [15]. Sodium is broadly consumed in cultures across the world as it improves the sensory properties of foods, principally by increasing saltiness, but also by decreasing any perceived bitterness [16], and may also interact with other flavors [17]. Thus, reducing the NaCl content of foods can significantly reduce palatability [18]. For this reason, food companies for many decades have been investigating flavoring agents that can replace some of the NaCl in foods without diminishing palatability, allowing consumers to reduce their sodium intake without losing out on flavor. Many NaCl substitutes have been investigated in the past, with potassium chloride (KCl) and monosodium glutamate (MSG) perhaps the most examined.

The average American consumes less than the recommended daily intake of potassium [19]. Since the incidence of many diseases associated with low potassium intake overlap with the incidence of diseases associated with high sodium intake [20], widespread potassium supplementation combined with sodium reduction in foods may benefit population health. MSG is the monosodium salt of glutamic acid, an amino acid naturally occurring in our bodies, and considered harmless despite historical (and arguably in some cases, hysterical) links to health conditions [21]. In animal studies, MSG intake has been linked with increased food intake and metabolic dysfunction [22], neurotoxicity [23], female infertility [24], and altered neuroendocrine function [25]. However, in a review of the literature on the potential health hazards of MSG, it was concluded that the previously reported negative health effects of MSG were based on dosing that greatly exceeded levels normally consumed in foods, and that there was little evidence supporting these claims in human dietary intake studies [21]. Given the evidence, MSG is a generally recognized as a safe (GRAS) substance by the U.S. Food and Drug Administration (FDA) [21].

The partial substitution of NaCl with KCl in foods such as rice, pizza crust, and dry-cured bacon has been reported to allow for a 25–40% sodium reduction with no difference in consumer liking (at the power tested) and no negative effects on sensory profiles [26,27,28]. Although KCl imparts saltiness, it may also provide off-flavors that limit the concentration to which it can be added to foods without diminishing palatability [29]. Most commonly, KCl is described as salty, but also bitter, and to a lesser degree, chemical and metallic [30]. Since NaCl suppresses bitterness in foods [16], the bitterness of KCl is a characteristic that must be overcome to realize its full potential as a sodium replacement.

MSG is used commercially in foods as a flavor enhancer, as it elicits a favorable taste of umami [31]. Previous research reveals that the partial substitution of NaCl with MSG in foods is possible without changing overall acceptability, reportedly allowing for up to a 32.5% reduction in sodium content [32]. Furthermore, at certain concentrations, added MSG in foods can not only enhance salty and sweet taste [33] but can also suppress bitterness [34]. Thus, MSG may, as well as tasting salty, be an effective flavoring agent to mask the off-flavors of KCl when used in sodium replacement, despite itself containing some sodium. For instance, it was reported that the addition of MSG could mask the unpleasant sensory attributes associated with partial NaCl replacement with KCl in fermented sausages, allowing for a 68% reduction in sodium while maintaining good consumer acceptance [35], although other flavor enhancers were also employed in this study. Thus, previous research on partial NaCl replacement with KCl or with MSG in foods supports the efficacy of these replacements individually in enhancing saltiness; however, few studies have investigated the influence of mixtures of NaCl, KCl and MSG on the sensory perception of real, complex foods. Further, perhaps fewer studies have evaluated consumer opinions on foods containing NaCl, KCl and MSG to assess whether the appeal of foods flavored in this manner differs from that of foods flavored with NaCl alone. Despite being naturally present in many foods, and its benefits as a flavor enhancer, in the United States the usage of MSG in foods still remains controversial. Wang and Adhikari (2018) found that the majority of consumers who completed a survey perceived MSG to be artificial and would prefer to consume foods containing no added MSG versus those containing added MSG [36]. Consumer perception of KCl in food is even more poorly characterized.

The aims of the current study were to: (1) determine the optimal concentrations of KCl and MSG in a reduced-sodium NaCl solution to most closely replicate the sensory profile of a regular NaCl solution, (2) assess consumer attitudes towards reduced-sodium foods containing KCl and MSG, and (3) ascertain whether a real finished food product, soup, flavored with these optimal concentrations of NaCl, KCl and MSG could maintain a degree of liking versus those flavored with typical concentrations of NaCl. To achieve these goals, we used descriptive sensory analysis, conjoint analysis, and consumer sensory testing, respectively.

## 2. Materials and Methods

### 2.1. Descriptive Analysis

#### 2.1.1. Panelists

The descriptive sensory study was carried out at the Department of Food Science at Cornell University. Eight trained descriptive panelists taking part in the study (five female and three male) had more than 40 h of training in descriptive sensory profiling. The panelists underwent retraining sessions in which astringent and metallic sensations were emphasized, along with salty, bitter and umami tastes. Panelists were trained with standard solutions of food grade alum (0.00148–0.00580 M), ferrous sulfate (0.00046–0.00178 M), NaCl (0.075–0.15 M), quinine (0.00023–0.00154 M), and MSG (0.01035–0.0414 M), for the sensations listed, respectively. The concentrations were based on standards accepted by the International Organization for Standardization and an optimized version from a previous study [37,38]. The definition for the terminology of each taste (slight, definite, pronounced) and the corresponding quantitative ratings on a 100-point line scale (30, 60, and 90, respectively) were discussed and agreed upon by all the panelists during the training. All panelists received compensation for completion of both training and evaluation sessions.

The panelists were instructed to complete the sensory evaluation of the samples in a quiet, distraction-limited location (a central location was not possible due to COVID). The panelists were instructed to name the location at which they completed the sensory testing using a location confirmation questionnaire via Redjade Sensory software (RedJade Sensory Solutions, Martinez, CA, USA). Sensory testing was divided over six sessions on three days with two sessions per day. Each session consisted of the evaluation of six or seven samples. All samples were assessed in blind duplicate, with all panelists evaluating the same six or seven samples two times in the same day, a single time per session. To prevent fatigue, the two daily sessions were separated by at least one hour. All samples were coded with a three-digit code and the evaluation order was counterbalanced. Panelists were instructed to evaluate the samples in the order instructed, and to cleanse their palate with water between samples. To determine the sensory properties of the solutions, participants were asked to rate the intensities of the saltiness, bitterness, umami, metallic and astringent flavors using an identical 100-point line scale as the one used during the training session via Redjade Sensory software.

#### 2.1.2. Samples

Food grade standards of all-purpose sodium chloride (Morton Salt, Inc., Chicago, IL, USA), potassium chloride, and monosodium glutamate (McCormick Culinary, Hunt Valley, MD, USA) were purchased from online retailers (Ingredi.com, Baltimore, MD, USA & Amazon.com, Washington, DC, USA). All of the solutions were prepared with deionized water. The samples consisted of 20 solutions of various NaCl, KCl, and MSG concentrations, along with NaCl only, KCl only, MSG only and water controls. The samples differed in the percent sodium difference from the high-NaCl control (Table 1). Concentrations of the salts were chosen to represent a range of NaCl levels (none, regular sodium and reduced sodium), KCl levels (none, low, medium, and high), and MSG levels (none, low, medium and high).

Regular-NaCl control sample levels (0.15 M NaCl) were selected to be comparable to those of a commercially available low-sodium soup according to previously published literature [39]. Other concentrations were chosen based on prior research by Sinopoli & Lawless (2012) who investigated the taste properties of various mixtures of KCl and NaCl in water [30], and by Jinap et al. (2016), who determined that an optimum level of MSG replacement for reduced-sodium soups was 0.7% (~0.0414 M) [32]. Twenty solutions for sensory evaluation were prepared and refrigerated one day prior to when they were to be evaluated. On the days of evaluation, the samples to be assessed were packaged into 2oz cups with lids and allowed to sit at room temperature for one hour before being provided to the panelists, who swished in the whole mouth, expectorated and rated the samples as above.

#### 2.1.3. Statistical Analysis

The results of the sensory evaluation were analyzed using analyses of variance (ANOVA) and post hoc Tukey’s tests, to determine significant differences among the means for saltiness intensity at the level of *p* ≤ 0.05 using Graphpad Prism statistical software (Dotmatics.com, Boston, MA, USA). Additionally, surface plots and radar plots were developed to show the relationships between NaCl, KCl, and MSG concentrations and attribute ratings using Microsoft Excel (Microsoft Corp, Redmond, WA, USA).

### 2.2. Conjoint Analysis

#### 2.2.1. Participants

All human subject research here and below was reviewed and approved by the Cornell University Institutional Review Board for Human Subject Research. Data from 150 participants was included in the analysis, with those marked as low quality or incomplete responses excluded. Participants were recruited through email, various forms of social media, and social networks among students, faculty and staff from Cornell University.

Participants who completed the survey had the option to enter a draw to be randomly chosen to receive $5 via a digital gift card service (Tango Card, Seattle, WA, USA) as compensation for completion of the survey.

#### 2.2.2. Procedure

**Figure 1:**
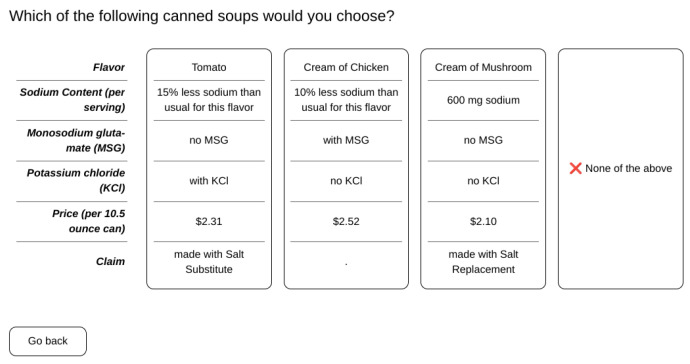
Example of theoretical canned soup options presented to participants.

The study was conducted using an online survey platform (Conjointly, Sydney, NSW, Australia). First, participants were asked to answer questions for Conjoint Analysis (CA). The options presented to participants in the CA design were descriptions of theoretical canned soups with differing levels of various attributes. The canned soup options differed in six attributes with each having a number of levels (Table 2). Choices presented to participants were three theoretical canned soup options of different combinations of all attributes and all levels. There was also an option of “None of the above”. One combination of the choices was presented to the participants is shown in Figure 1.

Flavor options were chosen based on common, high-selling soup flavors. It was assumed that flavor would be of high importance when selecting soup, but nonetheless this was included as an important part of the purchasing choice, and a presumed high-bar with which other factors could be compared. Sodium content was selected based on the average amount of sodium in one serving of soup [40] along with associated terms expressing lower levels than this, to understand whether consumers perceived overall sodium amount and percent sodium reduction differently. Prices were reflections of the average cost of a 10.5 ounce can of soup from multiple-surveyed online retailers (Amazon.com, Walmart.com), with claims chosen based on public opinion about salt substitutes and the most beneficial way to describe them.

Next, participant profiles were developed based on their responses to a series of phrases used to assess their attitudes about general health, sodium intake, and potassium intake. The phrases were adapted from Pires, de Noronha & Trindade, 2019, who investigated consumer perception of bologna with reduced sodium content and/or omega-3 enrichment [41]. A 7-point Likert scale was presented for consumers to define their degree of disagreement or agreement with a series of phrases, ranging from strongly disagree to strongly agree [42]. The presentation of phrases to the consumers was randomized. The phrases, separated by theme, used in this block are listed in Supplemental Table S1. Consumers were also asked how often they consumed canned soup, and how much they liked each flavor of the soup included in the CA questions, presented as a 7-point Likert scale [42]. Presentation of these questions about flavor were randomized. Lastly, participants were asked to provide information about their demographics. Analysis was carried out as above.

### 2.3. Consumer Study

#### 2.3.1. Panelists

The 101 panelists (mean age 33; 72% female, 27% male, 1% gender nonconforming) were untrained. Sensory evaluation sessions were held over two days, with each panelist completing the sensory evaluation only once. Panelists were recruited from the Cornell University campus and from the surrounding community (Ithaca, NY, USA). At the time of the study, the panelists were 18 years or older and had no intolerances to the ingredients of the chicken stock or to NaCl, KCl or MSG. Sensory testing was carried out in individual sensory booths at the Sensory Evaluation Center of the Department of Food Science at Cornell University. Panelists were asked to rate their overall liking of the samples using the 9-point hedonic scale, ranging from “dislike extremely” to “like extremely”. The panelists were also asked to rate the intensities of salty, umami, bitter, metallic, astringency, and a rating of overall aftertaste intensity from the samples using a 100 point generalized labeled magnitude scale (gLMS). The gLMS scale ranged from “no” to “strongest imaginable” sensation with verbal descriptors assigned to different levels of intensities in between [43]. All sensory data were collected with Redjade sensory software.

#### 2.3.2. Samples

NaCl, MSG and KCl were obtained as above. Unsalted chicken stock (Kitchen Basics, McCormick & Company, Inc., Hunt Valley, MD, USA) was purchased from an online retailer (Amazon.com, Washington, DC, USA). To prepare the stock soup, the unsalted chicken stock was diluted to 35 mg sodium per cup with filtered water. The soup stock was prepared in advance and refrigerated. The soups for sensory evaluation were flavored with differing levels of NaCl, KCl, and MSG to represent the no salt control, the high-sodium NaCl-only control, the low-sodium NaCl-only control and the two optimum levels of partial KCl and MSG replacement as determined by the descriptive analysis results (Table 3). The soups for sensory evaluation were prepared from the stock and refrigerated one day prior to the sensory evaluation sessions. 50 mL of each sample was served in 150 mL cups at 40 °C [32]. The temperature was kept constant by keeping soups in conductive containers in a water bath with a sous-vide circulator. All samples were coded with a three-digit number and the evaluation order was counterbalanced. The panelists were presented with the samples one at a time, and instructed to evaluate the samples in the order provided, and cleanse their palate with water between evaluating samples.

#### 2.3.3. Statistical Analysis

The results of the consumer evaluation were analyzed using repeated-measures analyses of variance (ANOVA) and post hoc Tukey’s tests, to determine significant differences among the means at the level of *p* ≤ 0.05, as above.

## 3. Results and Discussion

### 3.1. Sensory Optimization of Salty Taste with KCl and MSG

**Figure 2:**
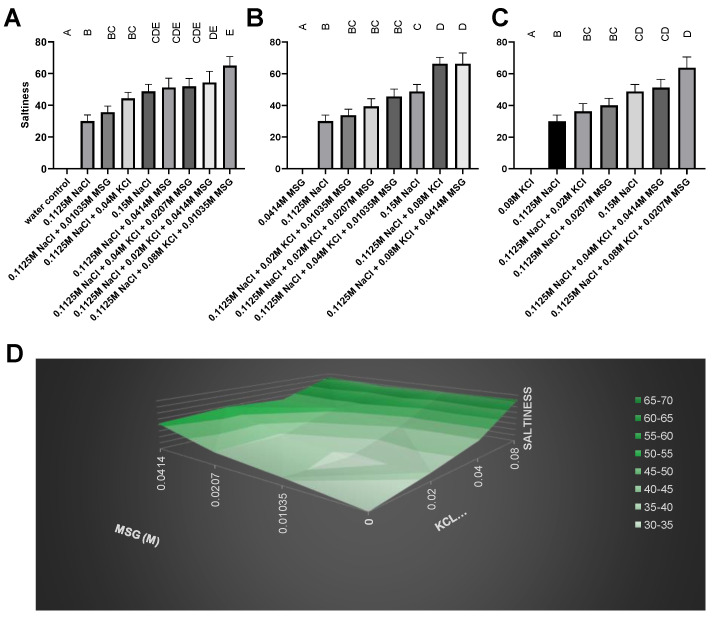
(**A**–**C**) Descriptive sensory scaling of saltiness from 3 sessions, measures in duplicate, bars denote mean and SEM, different letters denote statistical difference between columns. (**D**) Three-dimensional plot of perceived saltiness (*y*-axis) with ascending MSG (*x*-axis) and KCl (*z*-axis).

The trained descriptive sensory panel examined multiple concentrations of KCl and MSG in water, and in reduced-sodium (0.1125 M) solutions, versus regular-sodium (0.15 M) control samples. Interestingly, several samples, specifically those with high KCL, were perceived by the trained panel as statistically more salty tasting than the high NaCl sample (Figure 2). This is in contrast to work from Rodrigues and colleagues, who using KCl and MSG in combination in mozzarella cheeses reported that even though liking could be maintained, sufficient levels of saltiness could not [44].

Aside from saltiness, the descriptive panel also scaled bitterness, umami, metallic and astringent sensations arising from the samples, shown in Supplemental Figure S1. Only the sample high in KCl showed significant bitterness, with no sample significantly higher for astringency or metallic sensation. As expected, the samples containing MSG were rated as more umami than salt controls, and thus would only be suitable for use in food systems with native umami flavor; however, going forward, we chose to avoid such high concentrations of KCl, and sought first to optimize flavor profiles in a neutral base before going to a complex product, as some have [32,45], to make results more generalizable.

### 3.2. Consumer Attitudes to Sodium Reduction

**Figure 3:**
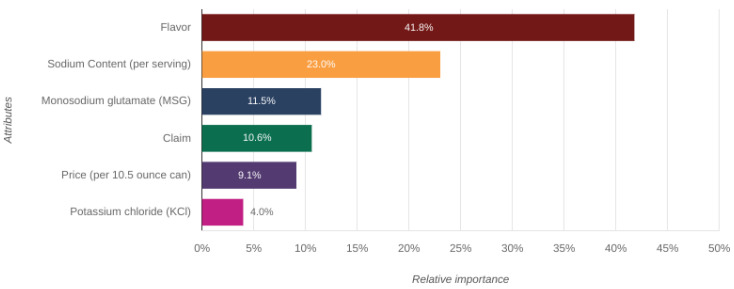
Perceived importance of each attribute in the selection of canned soups, relative to the other attributes.

Conjoint analysis is a form of choice experiment designed to implicitly gauge the value a consumer puts on an aspect of a product, as well as to aid in the optimization of an ideal version of said product [46]. Panel demographics can be found in Supplemental Table S2. For participants (*n* = 150), as expected, flavor was the most important attribute in the selection of canned soups (Figure 3). Sodium content was valued as slightly more than half as important as flavor, which illustrates the salience of sodium in the nation’s food choices. The participants attributed the lowest importance to KCl content.

**Figure 4:**
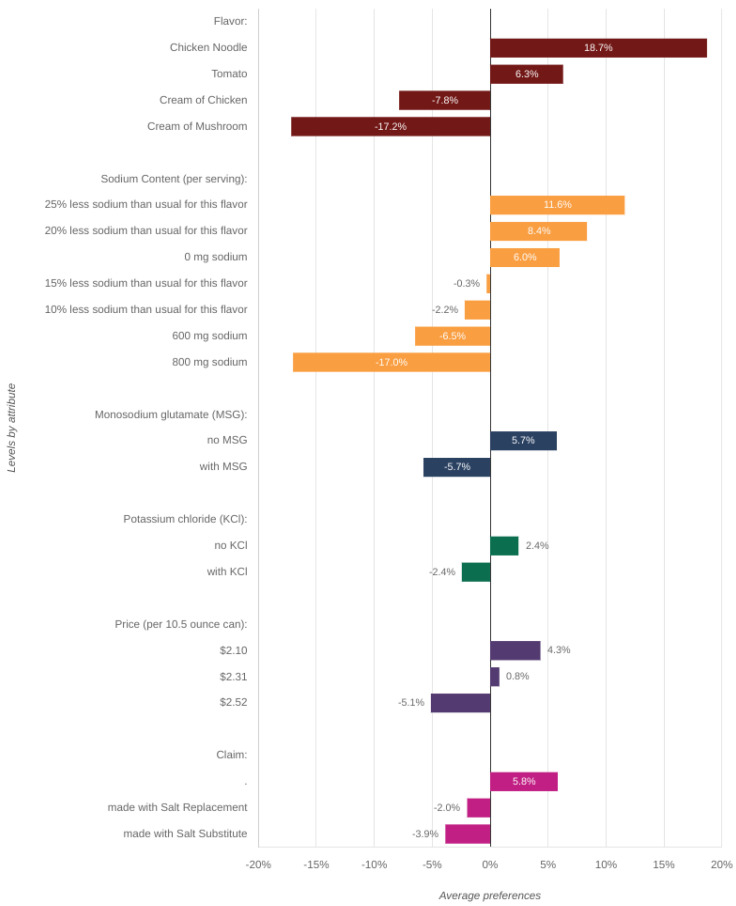
Relative preference for each level within an attribute in the selection of canned soups.

The participants ascribed similar importance to MSG content, claim, and price, which were all less important than flavor and sodium content, but more important than KCl content. Figure 4 shows the relative preference for each level within an attribute in the selection of canned soups. Positive values represent levels that were preferred by consumers and negative values represent levels that performed poorly relative to other levels. Regarding flavor, participants preferred chicken noodle and tomato soups, while not favoring cream of mushroom or cream of chicken soups. Participants favored greater percent reductions of sodium content (25 and 20%) and showed an aversion to lower percent reductions (15 and 10%). Participants did not favor being informed of the absolute amount of sodium in milligrams in their soups, preferring a percent reduction. This implies that consumers may not be aware of the sodium content in their foods to begin with. Even though 600 mg of sodium is approximately a 25% reduction for many soups [40], participants preferred to hear this as a percent reduction, rather than the absolute amount, and in fact were willing to consume canned soup with 0 mg of sodium, practically impossible, again showing they may not be aware of sodium content in the foods they select. Participants preferred no MSG over included MSG and also preferred no KCl over included KCl, and preferred less expensive cans over more expensive cans. Lastly, participants did not favor product claims about sodium replacements and substitutes, and favored canned soups that had no claims about sodium content, suggesting silent changes may be more beneficial in maximizing uptake of reduced sodium products than those where interventions are explicit. This is in agreement with reports that products labeled low salt may be perceived as less salty and/or less liked, even when only labels and not ingredients are varied [47]; a result not just confined to soups [48].

### 3.3. Consumer Acceptance of Reduced Sodium Soups Using Salt Replacement

**Figure 5:**
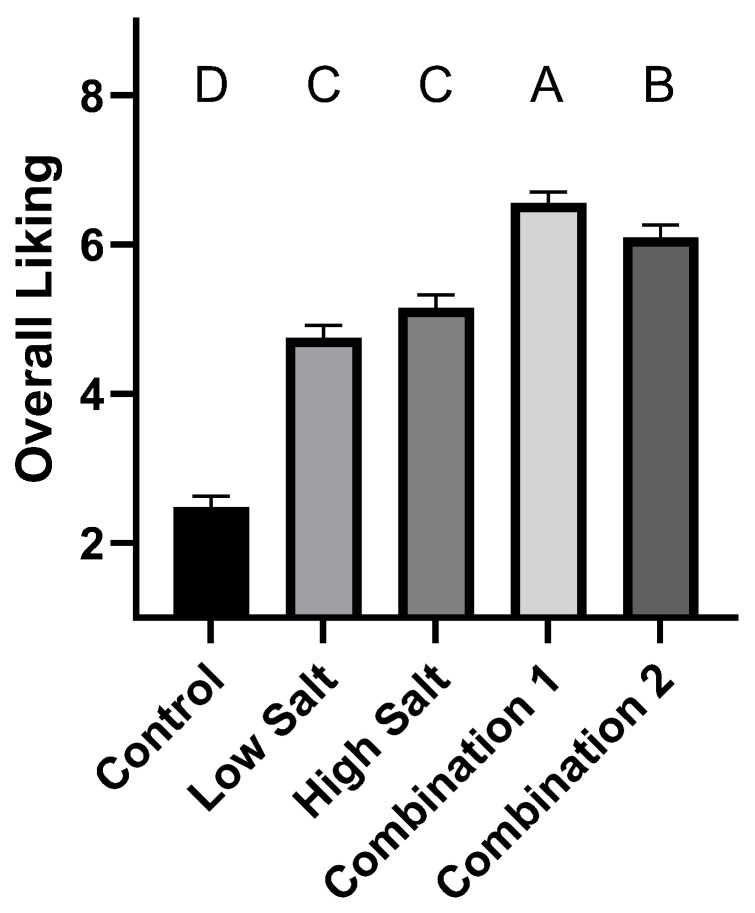
Overall liking from consumer panel of soup samples made with no added sodium, plus low and high salt, and low salt plus sodium replacement combinations 1 (30 mM KCl + 15.6 mM MSG) and 2 (60 mM KCl + 7.8 mM MSG). Bars show mean plus SEM. Different letters signify statistical differences between treatments.

Consumers assessed soups made from low sodium broths, seasoned with either no added seasoning (control), with 25% reduced sodium from the recommended seasoning (low salt; 84 mM NaCl), with the recommended amount of sodium for the broth (high salt; 113 mM NaCl), or low salt along with a combined sodium replacement strategy favoring MSG (combination 1; 30 mM KCl + 15.6 mM MSG) or favoring KCL (combination 2; 60 mM KCl + 7.8 mM MSG). Consumers rated the no-salt added broth as being the least liked (Figure 5), with low and high salt more highly liked, but high salt only trending higher than low, and not significantly different from one another. Both sodium replacement strategies were liked more than the high, low or no-added salt soups, with the strategy favoring MSG the most liked of any sample. KCl as a sodium replacement alone was reported by Lee et al. [49] to not always be sufficient to maintain liking in reduced sodium soups, suggesting MSG in combination with KCl may be a more effective strategy, as we saw in our test.

**Figure 6:**
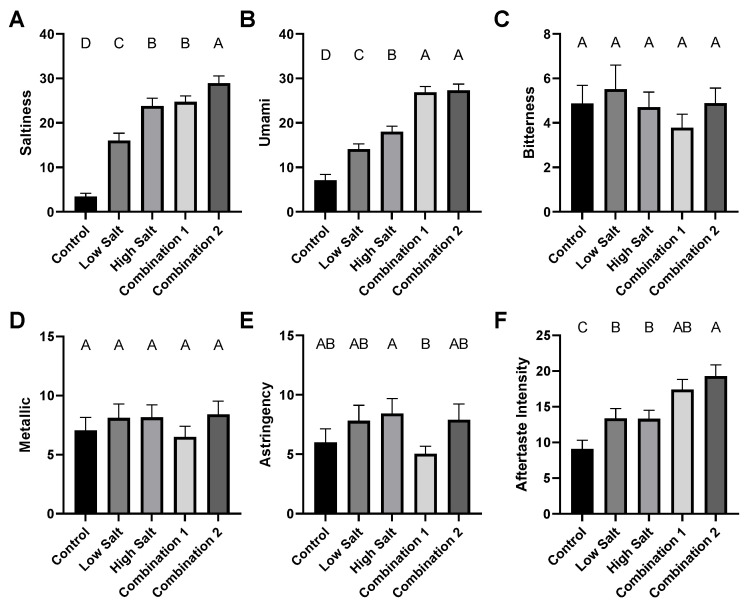
Scaling of intensities of saltiness (**A**), umami (**B**), bitterness (**C**), metallic flavor (**D**), astringency (**E**) and intensity of aftertaste (**F**) from consumer panel of soup samples made with no added sodium, plus low and high salt, and low salt plus sodium replacement combinations 1 (30 mM KCl + 15.6 mM MSG) and 2 (60 mM KCl + 7.8 mM MSG). Bars show mean plus SEM. Different letters signify statistical differences between treatments.

After rating their overall liking of the soups, the untrained consumers rated the intensity of saltiness (Figure 6A), umami (Figure 6B), bitterness (Figure 6C), metallic (Figure 6D), astringency (Figure 6E) and intensity of aftertaste (Figure 6F) of the soups. Results suggest that the sodium replacement strategies resulted in a more salty-tasting sample than the low salt sample (containing the same amount of NaCl), as well as a similar level of saltiness for combination 1 as the high salt sample, with a significantly higher saltiness for combination 2 than this higher sodium sample. This confirms that in this food system, sodium is not in itself singular in providing salty taste, and that some sodium can be replaced in soups with KCl and MSG, without a reduction in saltiness or liking. Importantly, these added salts did not increase perceived bitterness, metallic tastes or astringency in the soups, frequently cited off-flavors associated with sodium replacement. Although Hooge and Chambers [50] found bitterness in sodium-reduced soups with KCl with little difference from controls, no liking ratings were reported, with researchers reporting only descriptive recordings. The umami taste from the soups was higher for both combinations of sodium replacements than low or high salt soups alone, and the total perceived aftertaste was higher than both for combination 2; however, neither of these sensations are implicitly perceived as an off-flavor, and may in this system have even been positive to the panelists. There are also reports of enhanced cortical activity arising from the use of MSG in sodium reduction, which is not present with KCl alone, and seemed to be related to higher liking [51]. We would conclude that removing NaCl from foods without accounting for a loss of appetitive salty taste is not a realistic approach to reducing sodium intake, with consumers not likely to select foods that are liked less, or even perceived to be likely to lack the salty flavor they desire. Sodium reduction in soups was estimated in a modeling approach by Bruins [52] to have the potential to reduce incidences of stroke, AMI, angina and heart failure, alongside a reduction in burden of disease of around 800 lifetime disability-adjusted life years, highlighting the potential for health benefits from this relatively inexpensive strategy.

### 3.4. Limitations

There are some limitations to the present study. First, we did not analyze sex differences for the rating of attribute intensities or hedonic liking. Specifically regarding the consumer test, there was an overabundance of female participants relative to male participants. Previous research reported sex differences in taste processing, with females rating bitterness more strongly [53] and showing lower recognition thresholds for salty and bitter tastes [54]. However, it is important to note that one study reported no difference in the intensity perception of any individual taste between the sexes [55]. Furthermore, women who are pregnant may show changes in taste perception related to bitter and salty stimuli [56]. It is possible that the large proportion of female participants skewed the data to show higher intensities of bitterness and saltiness than would have been reported with a more even number of female and male participants. Despite this, the findings on whether there are sex differences in taste that lead to differing preference patterns in humans are inconclusive [57], thus, differences in individual attribute perception may have had limited influence on the overall liking of the soup samples. Second, the present study did not analyze age differences for attribute intensity ratings or hedonic liking. A review of the literature investigating the relationship between age and taste reported that taste perception declines with age [58]. The mean age of participants in the consumer test was 33 years. Given the evidence, we might expect lower intensity ratings for the attributes in older participants when compared with younger participants, but whether this might lead to alterations in preference remain speculative. Thirdly, we did not investigate the influence of body mass index (BMI) on attribute intensity ratings. It has been found that taste perception of saltiness [59] and umami [60] declines with increasing BMI, and that taste and obesity may be linked in animal and human studies [61,62]. Further, we did not check for hormonal modulation differences, which are also known to affect the gustatory system [63]. We also do not eliminate the possibility that some interaction between sensations of umami natively arising from soup samples may have altered our flavor profiles generated from the initial descriptive testing that took place in aqueous samples.

## 4. Conclusions

Sodium content is a concern for the modern food consumer. Despite this, consumers do not readily sacrifice taste for long-term health benefits from food. Our study highlighted that MSG and KCL can be used in combination to replace some of the sodium in foods without a decrease in perceived saltiness from these foods, or a drop in liking. Despite these benefits, consumers were not comfortable with these ingredients being highlighted in marketing, suggesting that a silent change may be more efficient in sodium-reduction strategies.

## Figures and Tables

**Table 1:** Solutions used and sodium difference from high-NaCl control.

Solution	NaCl (M)	KCl (M)	MSG (M)	%Na Difference
1	0.15	0	0	0
2	0.1125	0	0	−25
3	0	0	0	−100
4	0.1125	0	0.01035	−18.77
5	0.1125	0.02	0.01035	−18.77
6	0.1125	0.04	0.01035	−18.77
7	0.1125	0.08	0.01035	−18.77
8	0.1125	0	0.0207	−12.53
9	0.1125	0.02	0.0207	−12.53
10	0.1125	0.04	0.0207	−12.53
11	0.1125	0.08	0.0207	−12.53
12	0.1125	0	0.0414	0
13	0.1125	0.02	0.0414	0
14	0.1125	0.04	0.0414	0
15	0.1125	0.08	0.0414	0
16	0.1125	0.02	0	−25
17	0.1125	0.04	0	−25
18	0.1125	0.08	0	−25
19	0	0.08	0	−100
20	0	0	0.0414	−75.07

**Table 2:** Attributes and levels investigated in conjoint analysis.

Attributes	Levels
Flavor	Chicken Noodle
	Cream of Chicken
	Cream of Mushroom
	Tomato
Sodium content (/serving)	0 mg sodium
	600 mg sodium
	800 mg sodium
	25% less sodium than usual for this flavor
	20% less sodium than usual for this flavor
	15% less sodium than usual for this flavor
	10% less sodium than usual for this flavor
MSG	no MSG
	with MSG
KCl	no KCl
	with KCl
Price (/can)	$2.10
	$2.31
	$2.52
Claim	none
	made with salt replacement
	made with salt substitute

**Table 3:** Sample added salt concentrations and sodium difference from high-sodium NaCl control. Levels do not include salts already present in broth.

Solution	NaCl (mM)	KCl (mM)	MSG (mM)	% Na Difference
Unsalted Control	0	0	0	−94.66
Low-sodium NaCl	84.375	0	0	−23.67
High-sodium NaCl	112.5	0	0	0
Combination 1	84.375	30	15.525	−11.87
Combination 2	84.375	60	7.7625	−17.78

## Data Availability

Data available on request.

## Supplementary Materials

The following supporting information can be downloaded at: https://www.mdpi.com/article/10.3390/foods12102063/s1, Figure S1: Radar plot of descriptive data; Table S1: Phrases used in attitudinal questionnaire separated by theme; Table S2: Panel demographics.
