# Supplementary material for: Sodium Replacement with KCl and MSG: Attitudes, Perception and Acceptance in Reduced Salt Soups
Source: Foods. 2023 May 20;12(10):2063. doi: 10.3390/foods12102063 (PMC10217644; doi:10.3390/foods12102063)
Supplement: Supplementary file 1 [file foods-12-02063-s001.zip › foods-2406202-supplementary.pdf]

Supplemental Material

**Attitudes about general health**
5. I am very concerned about how healthy foods are.
6. I would pay more for healthier canned soups.
7. I care about ingredients and read the labeling of processed foods.
**Attitudes about sodium intake**
8. For me, a diet low in sodium is important.
9. I would consume canned soup with reduced sodium content.
**Attitudes about potassium intake**
10. In my opinion, potassium intake can improve health.
11. I would pay for canned soup containing potassium.
12. For me, a diet adequate in potassium is important.

Supplemental Table S1: Phrases used in attitudinal questionnaire separated by theme

In which US region do you live?	%
North-east	76.0
South	6.7
Midwest	2.7
West	7.3
I don’t live in the US	7.3
Which of the following best describes your education level?	%
Primary school / Some high school	0.0
High school graduate	2.7
Other post-high school vocational training	0.7
Bachelor’s degree student	19.3
Bachelor’s degree graduate	28.0
Master’s degree student	4.7
Master’s degree graduate	24.7
Doctorate	18.0
Prefer not to say	2.0
What is your age group?	%
Under 18	0.0
18-24	30.0
25-35	37.3
36-45	10.0

46-55	4.0
56-65	9.3
Over 65	9.3
Which of the following best describes your racial/ethnic background?	%
White or Caucasian	56.7
American Indian or Alaska Native	0.7
Asian	25.3
Black or African American	8.7
Hispanic or Latino	8.0
Native Hawaiian or Pacific Islander	0.7
Other	3.3
I prefer not to answer	5.3
Please indicate how you identify in terms of gender	%
Male	18.7
Female	79.3
Non-binary	1.3
Prefer not to answer	0.7

Supplemental Table S2: Panel demographics

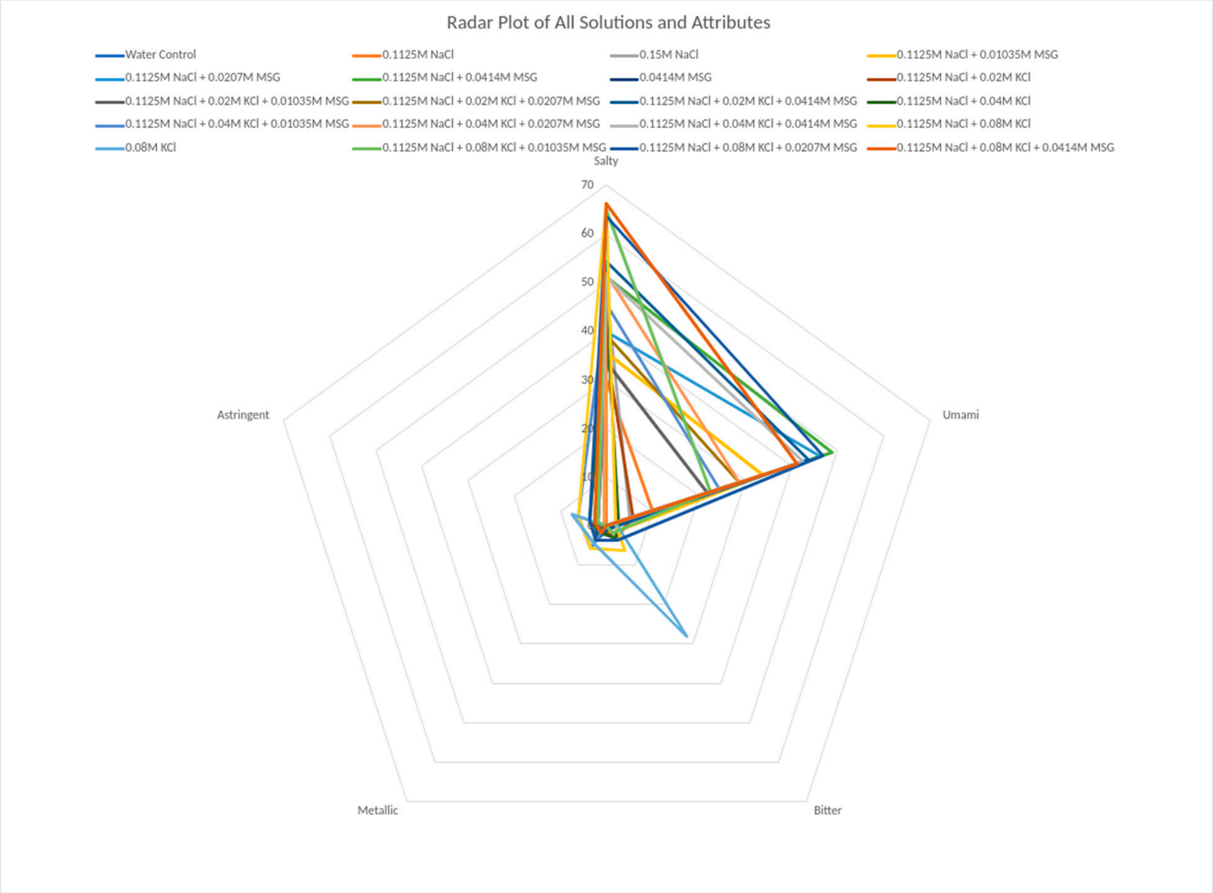

Supplemental Figure S1: Radar plot of descriptive data
